# Current employment characteristics and career intentions of Lithuanian dentists

**DOI:** 10.1186/1478-4491-12-74

**Published:** 2014-12-20

**Authors:** Vilija Janulyte, Jolanta Aleksejuniene, Alina Puriene, Vytaute Peciuliene, Habib Benzian

**Affiliations:** Institute of Dentistry, Faculty of Medicine, Vilnius University, Zalgiris st. 115, Vilnius, Lithuania; Faculty of Dentistry, University of British Columbia, 2199 Wesbrook Mall, Vancouver, Canada; College of Dentistry, New York University, 345 E. 24th Street, New York, USA

**Keywords:** Career intentions, Dentists, Emigration, Working conditions

## Abstract

**Background:**

The present survey explored the current employment profile and future career intentions of Lithuanian general dentists and specialists.

**Methods:**

A census sampling method was employed with data collected by means of a structured questionnaire that inquired about demographics, different employment-related aspects (practice type and location, working hours, perceived lack of patients, etc.), and future career intentions (intent to emigrate, to change profession, or the timing of retirement). The final response rate was 67.6% corresponding to 2,008 respondents.

**Results:**

The majority of all dentists work full or part-time in the private dental sector, more than one third of them owns a private practice or rents a dental chair. A minority of dentists works in the public dental sector. According to the survey, 26.6% of general dentists and 39.2% of dental specialists works overtime (>40 hours per week; *P* <0.001) and practice in multiple clinics (1.4 ± 0.6 and 2.0 ± 1.2, respectively; *P* <0.001). One third of general dentists (31.3%) and dental specialists (31.4%) stated to have a low number of patients (*P* >0.05). The majority (68.9% of general dentists and 65.9% of dental specialists) plans to work after the retirement age (*P* >0.05). Emigration as an option for their professional career is being considered by 10.8% of general dentists and 8.3% of dental specialists (*P* >0.05). Working either full or part-time in private practices (OR = 4.3) and younger age (≤35 years; OR = 2.2) are the two strongest predictors for a perceived insufficient number of patients.

**Conclusions:**

One third of dentists in Lithuania work long hours and lack patients. Many dentists practice in multiple locations and plan to retire after the official retirement age. Some dentists and dental specialists plan to emigrate. The perceived shortcomings within the dental care system and workforce planning of dentists need to be addressed.

## Background

The structure of professional dental care in Lithuania has gone through substantial changes since the country regained independence in 1991 [1]. During this time, dental care gradually and increasingly transitioned from a public and free-of-charge dental care system to a two-tier dental care delivery model including both private and public dentistry. Today, public dentistry provides free dental treatment for all children, whereas adults only have to cover the expenses for dental materials that are used for their treatment. Although Lithuania is categorized as a high-income country by the World Bank, with a GDP of USD 42.34 billion in 2012, it has recently been facing economic difficulties [2]. Against this backdrop, the government does not allocate sufficient monetary resources to offer free, good quality dental treatment for all citizens. This results in deficits in quality of care on the one hand and high patient co-payments on the other [3]. Voluntary health insurance plans just recently emerged in Lithuania. There are very few companies which provide voluntary dental insurance for their employees, and those seeking private dental care usually cover the treatment costs privately. About 41% of Lithuanian residents visit public dental institutions, 33% choose the private sector, and 26% visit both [3]. In 2014, the population of Lithuania amounted to 2.9 million residents, characterized by an ageing population structure [4, 5]. The prevalence of caries and periodontal problems is high when compared with other European countries [6, 7].

Training for dentists and dental specialists is offered at two universities in Lithuania. Dentists apply for their license after the 5-year undergraduate program. After another 3 years of postgraduate studies they can also acquire a dental specialist license. Six dental specialties are recognized in Lithuania, including oral surgeon, periodontologist, endodontist, prosthodontist, orthodontist, and pediatric dentist. About 150 dentists and 24 dental specialists have graduated annually over the last 3 years. Nevertheless, while the number of graduates in dentistry has been increasing by approximately one third during the last 3 years, the number of graduated dental specialists remained the same [8].

Since the country’s independence, the number of dentists in Lithuania has increased from an average of 5.5 dentists/10,000 inhabitants in 1992, to 6.5 dentists in 2002, and to 12.4 dentists in 2013 [9, 10]. As a comparison, the average for the European Union amounted to 6.7 dentists per 10,000 residents in 2012 [9]. Similar to other countries, a concentration of dentists is found in the big city areas of Lithuania [11–13]. A general tendency to an oversupply of dental professionals has been reported [14, 15]. The tense situation in the dental care market results in strong competition between colleagues and may be a push factor for emigration to countries with a shortage of dentists. International migration of dental professionals is a known phenomenon [16, 17]. In Lithuania, this may be reinforced because some European countries have abolished all restrictions for international health workforce migration [18]. This deregulation has opened new professional possibilities for Lithuanian dentists, particularly since Lithuania joined the European Union in 2004. During the economic crisis in 2010, the intentions to emigrate among general dentistry graduates amounted to 26.9% [15].

Analyzing different aspects of dentists’ current working environment is important in understanding the structure of the dental workforce, and its possible strengths and shortcomings. The aim of the present study was, therefore, to explore the current employment profile, working conditions, and future career intentions of Lithuanian general dentists and dental specialists.

## Methods

The study was approved by the National Data Protection Inspectorate (No. 2R-3247). An ethics approval was not required due to the nature of the study. Contact information (e-mail, address, telephone number) of all licensed dentists in Lithuania was acquired from the License Registry of the Lithuanian Dental Chamber in October 2012. The retired and emigrated dentists were excluded from the sample. The overall study sample consisted of all licensed dentists and dental specialists in Lithuania (n = 2,971). All dentists were contacted up to three times. Firstly, depending on the available contact information (e-mail or address), questionnaires were sent either electronically or by post. Non-responders received copies of the same questionnaire again after six weeks. Those who did not respond after the second time were contacted again by phone six weeks later and the questionnaire was re-sent via their preferred mode. In total, 2,008 questionnaires were returned and the final response rate was 67.6%. The data were collected from December 2012 to June 2013.

Reliability of the original study questionnaire was tested by asking 10 randomly chosen dentists to complete the questionnaire twice with a 2-month gap in between these recordings in order to avoid memory bias. The questionnaire items were structured on nominal, ordinal, and interval scales. The reliability of questions structured on nominal or ordinal scales was tested employing Cohen’s kappa and interval scale responses were checked by intra-class correlation. Overall, the reliability was high for questionnaire items falling within the range 0.7 to 1.0. The questionnaire included questions about demographic and employment-related characteristics such as practice location, practice type, employment status, number of work places, working hours, perception of insufficient numbers of patients, required additional workload, and future career intentions.

The SPSS statistical program version 21.0 was employed for all statistical analyses. Univariate analyses were used to describe the study sample regarding demographic characteristics and some study variables. The bivariate analyses were done for the following purposes: non-response analyses (χ^2^ test/Fisher test and independent samples *t*-test), comparisons between general dentists and dental specialists regarding demographic characteristics and concerning different aspects of their employment and future professional plans (χ^2^ test/Fisher test), and to assess a number of determinants in relationship to perceived low numbers of patients for all dentists and dental specialists (χ^2^ test/Fisher test). The multivariate logistic regression model with Enter method was used to assess the joint effect of determinants related to perceived low numbers of patients. The threshold for significance for all tests was set at *P* <0.05. Due to some missing answers for individual questions of the questionnaire, the statistics for each question were based on a varying number of study subjects.

## Results

The analyses showed no significant differences between responders and non-responders regarding the number of different dental specialists (oral surgeons, periodontologists, endodontists, prosthodontists, orthodontists, and pediatric dentists; *P* = 0.252). However, there were significantly fewer younger dentists (*P* = 0.001), males (*P* < 0.001), and dentists from big cities (*P* < 0.001) among the responders compared to the non-responders (results are not presented).

Table 1 describes socio-demographic and employment-related characteristics of Lithuanian general dentists and dental specialists. There were statistically significant differences between the two professional groups regarding gender distribution, residence, practice location and type, and working hours (*P* < 0.05). The group of dental specialists comprised significantly more males than the group of general dentists. More specialists, as compared to general dentists, tended to work in cities and overtime (>40 hours per week). In both professional groups, the majority of dentists worked full or part time in private practices and less than 25% of all dentists were employed in public clinics. The majority of both groups were associate dentists. More than one third of dentists owned a private practice or rented a dental chair. Both general dentists and dental specialists practiced in multiple clinics. Specialists practiced in significantly more employment sites compared to general dentists (2.0 ± 1.2 and 1.4 ± 0.6, respectively; *P* < 0.001).

**Table 1:** **Socio-demographic and employment-related characteristics – comparison between general dentists and dental specialists**

Demographic and employment-related characteristics	General dentists		Specialists		Total		***P***values
	n	%	n	%	n	%	
Sex*							
Males	198	12.0	103	29.9	301	15.1	<0.001
Females	1,453	88.0	242	70.1	1,695	84.9	
Total	1,651	100	345	100	1,996	100	
Age groups*							
35 years or less	589	35.7	109	31.6	698	35.0	0.183
36–55 years	592	35.9	141	40.9	733	36.7	
56 or more years	470	28.5	95	27.5	565	38.3	
Total	1,651	100	345	100	1,996	100	
Residence*							
Big cities	1,082	65.7	284	82.3	1,366	68.6	<0.001
Suburban or rural	565	34.3	61	17.7	626	31.4	
Total	1,647	100	345	100	1,992	100	
Practice location*							
Big cities	907	57.1	243	70.4	1,150	59.5	<0.001
Suburban or rural	681	42.9	102	29.6	783	40.5	
Total	1,588	100	345	100	1,933	100	
Practice type*							
Public	373	22.8	54	15.8	427	21.6	<0.001
Public and private	326	19.9	122	35.7	448	22.6	
Private	939	57.3	166	48.5	1,105	55.8	
Total	1,638	100	342	100	1,980	100	
Employment status*							
Associate dentist	1,016	63.3	211	62.1	1,227	63.1	0.154
Owns private practice	413	25.7	101	29.7	514	26.4	
Rents a dental chair	176	11.0	28	8.2	204	89.5	
Total	1,605	100	340	100	1,945	100	
Working hours (per week)*							
Part-time (≤30 hours)	470	28.9	89	26.3	559	28.4	<0.001
Full-time (30–40 hours)	725	44.6	117	34.5	842	42.8	
Overtime (>40 hours)	432	26.6	133	39.2	565	28.8	
Total	1,627	100	339	100	1,966	100	

*χ^2^ test.

Table 2 compares the two groups of dentists concerning their future career intentions. No statistically significant associations were found. Almost 11% of general dentists and 8.3% of dental specialists considered emigration as an option, and a small proportion of dentists contemplated changing professions. Relatively high proportions in both groups of dentists (>65%) had plans to work after retirement age should their health allow it. The retirement age in Lithuania is progressively increasing, and is currently set at 61 years for women and 63 years for men [19].

**Table 2:** **Future career intentions of general dentists and dental specialists**

Future carrier intentions	General dentists		Specialists		Total		***P***values
	n	%	n	%	n	%	
Intention to emigrate*							
Yes	175	10.8	28	8.3	203	10.3	0.202
No	1,450	89.2	310	91.7	1,760	89.7	
Total	1,625	100	338	100	1,963	100	
Intention to change profession*							
Yes	42	21.0	6	1.8	48	8.9	0.445
No	158	79.0	334	98.2	492	91.1	
Total	200	100	340	100	540	100	
Intention to retire*							
Earlier than retirement age	93	5.8	30	8.9	123	6.3	0.096
At retirement age	407	25.3	85	25.2	492	25.3	
Continue working at retirement age	1,109	68.9	222	65.9	1,331	70.2	
Total	1,609	100	337	100	1,946	100	

*χ^2^ test.

**Figure 1:**
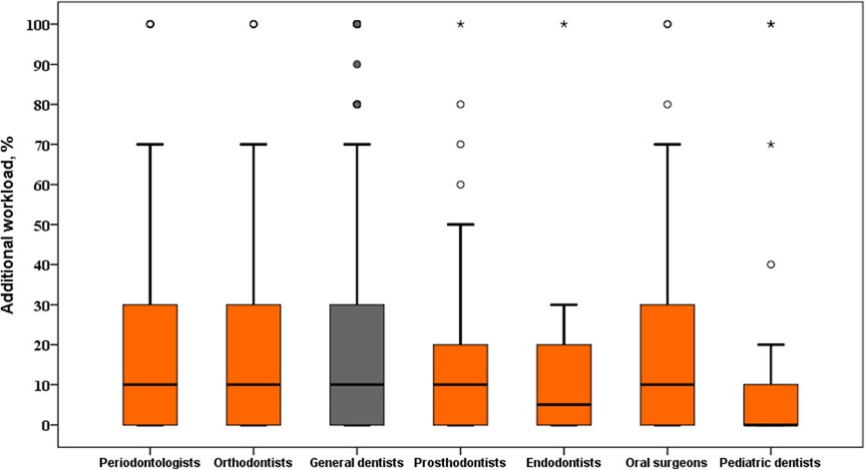
**The distribution of required additional workload among dentists and different dental specialists.**

Table 3 presents the results of bivariate analyses where a number of determinants were associated with perceived low numbers of patients. Around one third in both groups of dentists reported a low number of patients. More males (*P* = 0.001) and younger dentists (*P* < 0.001) stated insufficient numbers of patients compared to their female and older professional counterparts. Urban dentists lacked patients slightly more than dentists from suburban or rural areas (*P* < 0.001). Dentists working full or part time in private practices perceived to have insufficient numbers of patients more often compared to those who practiced in the public sector (*P* < 0.001). Almost 16% of general dentists and dental specialists who perceived low patient numbers intended to emigrate and only 8.1% of dental professionals who did not indicate a lack of patients intended to do so. The difference was statistically significant (*P* < 0.001). There were no statistically significant differences between dentists’ perceived lack of patients and intended retirement time.Figure 1 shows the distribution of required additional workload among dentists and different dental specialists. Endodontists and pediatric dentists required the least additional workload. The average dentist, periodontist, orthodontist, prosthodontist, and oral surgeon required about 10% of the additional workload. However, about half of these professionals required more than that.

**Table 3:** **Determinants related to the perceived shortage of patients (all sample)**

Demographic, employment-related characteristics, and future carrier intentions	Lacking patients		Not lacking patients		Total		***P***values
	n	%	n	%	n	%	
Dental professionals*							
General dentists	515	31.3	1,128	68.7	1,643	82.7	1.000
Specialists	108	31.4	236	68.6	344	17.3	
Total	623	31.4	1,364	68.6	1987	100	
Gender*							
Males	120	39.7	182	60.3	302	15.1	0.001
Females	506	29.8	1,190	70.2	1,696	84.9	
Total	626	31.3	1,372	69.9	1,998	100	
Age groups*							
35 years or less	309	44.1	391	55.9	700	35.0	<0.001
36–55 years	202	27.5	533	72.5	735	36.8	
56 or more years	115	20.4	448	79.6	563	71.8	
Total	626	31.3	1,372	69.9	1,998	100	
Practice location*							
Big cities	402	35.0	747	65.0	1,149	59.5	<0.001
Suburban or rural	206	26.3	576	73.7	782	40.5	
Total	608	31.5	1,323	68.5	1,931	100	
Practice type*							
Public	48	11.2	381	88.8	429	21.6	<0.001
Public and private	172	38.2	278	61.8	450	22.6	
Private	404	36.4	705	63.6	1,109	44.2	
Total	624	31.4	1,364	68.6	1,988	100	
Intention to emigrate*							
Yes	97	15.7	109	8.1	206	10.5	<0.001
No	521	84.3	1,245	91.9	1,766	89.5	
Total	618	31.3	1,354	68.7	1,972	100	
Intention to change profession*							
Yes	16	2.6	32	2.4	48	2.4	0.77
No	605	97.4	1,325	97.6	1,930	97.6	
Total	621	31.4	1,357	68.6	1,978	100	
Intention to retire*							
Earlier than retirement age	41	33.1	83	66.9	124	6.3	0.894
At a retirement age	156	31.6	338	68.4	494	25.3	
Continue working at retirement	416	31.1	922	68.9	1,338	68.4	
Total	613	31.3	1,343	68.7	1,956	100	

*χ^2^ test.

Table 4 presents findings from the logistic regression analysis where the dependent outcome was the perceived low number of patients and the independent predictors were practice type, age, gender, practice location, type of dentist (general dentists or specialists), and intention to emigrate. The two strongest predictors associated with perceived low patient numbers were working in a private practice or in public and private practices combined (OR = 4.4, *P* < 0.001) and younger age (≤35 years; OR = 2.0, *P* < 0.001). The likelihood of low patient numbers was 1.5 times higher for males (*P* = 0.007) and 1.5 higher for those who practice in cities compared to those who practice in rural areas (*P* = 0.001). The perception of low patient numbers was 1.7 times more frequent for those who intend to emigrate (*P* = 0.01).

**Table 4:** **Multivariate analysis of determinants related to the perceived lack of patients (logistic regression)**

Predictors		B	Significance	Adj. odds ratio	95% CI for OR
Practice type	Private or private & public	1.478	<0.001	4.4	3.1; 6.2
	Public	0		1.0	
Age group	≤35 years	0.709	<0.001	2.0	1.7; 2.5
	>35 years	0		1.0	
Gender	Males	0.380	0.007	1.5	1.1; 1.9
	Females	0		1.0	
Practice location	Urban	0.368	0.001	1.5	1.2; 1.8
	Suburban and rural	0		1.0	
Type of dentist	Specialists	0.126	0.646	1.1	0.9; 1.5
	General dentists	0		1.0	
Intention to emigrate	Yes	0.551	0.010	1.7	1.3; 2.4
	No	0		1.0	
Constant		-2.755	<0.001	0.064	

## Discussion

The present study findings indicate that a proportion of Lithuanian dentists and dental specialists face a number of challenges. About one third of dentists work long hours, perceive an insufficient number of patients, and require additional workloads. Further, the majority of them practice in multiple locations. A large share of dentists also considered a continuation of work beyond the official retirement age. The challenges in the dental profession are also present in other EU countries: Greece has reported an oversupply and unemployment of dentists [20]. Unemployment of dentists is also an issue in Finland, Germany, and Italy [21].

The results show that the perception of having low numbers of patients was significantly higher among dentists working in a private practice. These results might indicate that there is an uneven distribution of dentists among the private and public sector that does not reflect the demand of patients for the respective services. According to Puriene et al. [3], patients in Lithuania, especially those with lower income, prefer services in the public sector as the treatment is less expensive. Patients who demand treatment with modern technologies tend to visit private dental clinics [3]. As dental plans are rare in Lithuania, only patients willing to pay more can access treatments at private practices.

The figure of the required additional workload among dental professionals might indicate a surplus of dentists and dental specialists in Lithuania. In 2013, 46.4% of all dentists were 40 years old or younger, while only about 17% of physicians, 24% of obstetricians, and 30% of nurses were in the same age group in 2010 [8, 22]. In Australia, 37.6% of dentists were 39 years old or younger. In the USA and Germany, 36% and 39% of dentists, respectively, were 44 years old or younger [23–25].

According to the study results, emigration intentions are expressed more often by those dentists who perceive a lack of patients. An increasing lack of patients may therefore result in an increasing number of dentists intending to emigrate to countries where there is demand for a professional dental workforce with better working conditions and career opportunities. On the one hand, this may lessen the competition between dentists and improve the problem with insufficient numbers of patients in areas where the dentist-patient ratio is high. On the other hand, emigration might lead to shortages in areas where the dentist-patient ratio is already low. Given the high costs of dental education, which are covered by the government, emigration may also be considered as a loss to the national economy. However, recent dentists’ intentions to emigrate are lower when compared to other specialties during the time of Lithuania’s accession to the European Union. In 2004, 26.8% of physicians and 26.5% of pharmacists reported an intention to emigrate [26, 27].

A closer look at possible underlying reasons for the perceived unequal distribution of patients among dentists practicing in the public and private sector reveals a lack of regulation of the dental workforce and its regional distribution. A few studies have analyzed the unequal distribution of dentists across Lithuania and emphasized the need for dental workforce planning [28–30]. However, no appreciable measures have yet been taken by policy makers and health care planners in Lithuania to address these shortcomings. In contrast to the dental care system, a similar situation in the general health care system was encountered actively. This comprised increasing student enrolments to medical studies to counterbalance the predicted shortage of physicians, addressing mal-distribution of specialized physicians by providing recommendations to universities, partly outweighing geographical mal-distribution by facilitating agreement between medical residents and health care institutions on covering medical residency costs as well as full or partial subsidizing costs [31]. As the shortcomings in the dental care system are acute, similar measures are required.

## Conclusions

Working conditions of Lithuanian dentists and dental specialists have been examined. Some of the challenges dentists and dental specialists face in Lithuania include long working hours, a perceived lack of patients, practicing in multiple locations, intention to continue working after official retirement age, and possible intention to emigrate. These indications should be further researched and analyzed to inquire the exact underlying causes, to identify shortcomings, and to inform and improve workforce planning. Especially the regulation of the number and regional distribution of practitioners needs to be addressed by policy makers and health care planners in Lithuania in a timely manner. This will be essential in order to balance demand for and supply of adequate and affordable dental care. The overall goal should be to ensure equitable access to oral care for all segments of the population in the country while at the same time allowing for economically sustainable working conditions for dentists, both in public service and private practice.
